# Food allergies and allergens: Characterization and perceptions among diagnosed food allergic individuals in Lebanon^☆^

**DOI:** 10.1016/j.waojou.2020.100481

**Published:** 2020-11-09

**Authors:** Hani Dimassi, Suzan Haidar, Sarah Issa, Hussein F. Hassan

**Affiliations:** a School of Pharmacy, Lebanese American University, 1102 2801, Koraytem, Beirut, Lebanon; b Department of Nutrition and Food Sciences, School of Arts and Sciences, Lebanese International University, Salim Salam, Beirut, Lebanon; c Department of Natural Sciences, Nutrition Program, School of Arts and Sciences, Lebanese American University, 1102 2801, Koraytem, Beirut, Lebanon

**Keywords:** Food allergy, Food allergens, Perceptions, Lebanon

## Abstract

**Background:**

The aim of our study was to assess the knowledge, practices, and attitudes towards food allergens and allergies among diagnosed food allergic individuals in Lebanon.

**Methods:**

Seventy diagnosed participants were recruited after reaching out to all the allergists of the country. They completed in person or over the phone a comprehensive valid questionnaire composed of 49 questions.

**Results:**

Wheat was reported as top food allergen (15.7%), while itchy skin and rash were the most reported symptoms (71.4% and 68.6%, respectively). Only 34 (48.6%) of participants indicated they carry medications, 58 (70.7%) were diagnosed using blood test, and 22 (31.4%) stated that they are very knowledgeable on the topic. In terms of knowledge, participants scored on average 84.2 ± 11.5%. In terms of best practices, participants scored on average 47.8 ± 28.3%. Having a health related educational background increased significantly (p < 0.05) both knowledge and best practices scores, while age and gender did not have an effect.

**Conclusions:**

Our results highlight the importance of organizing ongoing educational initiatives and emphasize the need to lobby policy makers to making allergen-warning labels obligatory in the country.

## Introduction

Food allergy is an issue of public health concern since it triggers life-threatening reactions. It is an adverse immune reaction to a food allergen, mainly of protein nature, and takes place when the allergic individual is exposed to the allergen, resulting in symptoms that are varied and can range from minor skin reactions to major anaphylactic reactions, possibly leading to mortality. Food allergy affects between 2% and 10% of the world population.^1^ According to the National Health and Nutrition Examination Survey (NHANES), the prevalence of food allergy was 9%, with a prevalence of 7% in children and 10% in adults. Atopic dermatitis, a family history of atopy, and asthma are the main risk factors to develop a food allergy.^2^ These risk factors have been documented by many studies suggesting that breakdown of the skin barrier in atopic dermatitis can result in epicutaneous sensitization to foods resulting in a food allergy.^3^ Obesity and Vitamin D deficiency have also been linked to increased risk of food allergies.^1^

Food allergies and food intolerances are 2 distinct conditions which have different diagnostic routes and are commonly confused by patients as being 1. Food intolerance, contrary to allergy, is a form of a non-immunologically mediated reaction. It is triggered by foods such as milk, rice, soy, oat, and rarely meat, and is associated usually with gastrointestinal symptoms, including diarrhea, abdominal pain, bloating, flatulence, and nausea, with no serious life threatening reactions. A common food intolerance is that of lactose intolerance where the inflicted individual is unable to produce adequate levels of the lactase enzyme necessary to digest lactose, often resulting in diarrhea and abdominal pain.^4^ One hundred seventy foods have been recognized as allergy-causing; however, only a small number of these foods are responsible for a majority of reactions. According to Codex, the big 8 common food allergens, which contribute by 90% of food allergic reactions worldwide, shall be declared. These include egg, milk, gluten containing products, soy, tree nuts, peanuts, fish, and shellfish.^5^

Avoiding food allergens is the main prevention and management strategy of food allergies, but sticking to an allergen-free diet is not easy, especially when consumers dine out or buy ready-made meals due to inappropriate labeling or hidden undeclared allergens, miscommunication, or cross contamination.^6^

Few studies on knowledge and practices related to food allergies among food handlers were conducted worldwide. There is still a scarcity of research in this field in developing countries, such as the Middle East and North Africa region, where no study tackled so far the diagnosed food allergic individuals.

## Methods

### Study population

Participants in our study were Lebanese adults above 18 years old, allergic to food, and diagnosed by a medical doctor (allergist, gastroenterologist, etc). Non-medically or self-diagnosed individuals were excluded. Our study was endorsed by the Lebanese Society of Allergy and Immunology, which provided us with the database of allergists in the country along with their contact info. The president of the society then sent them a WhatsApp message and email to brief them about our study and encourage them to assist us in our recruitment efforts. Then, we visited each and every allergist clinic and got their consent to go over the medical records and to identify medically diagnosed food allergic individuals. In addition, we sent an email to the faculty, staff, and students in our university asking them to identify any acquaintances whom we can contact to participate in our study. Furthermore, all investigators posted on their social media about the study. Eighty-two diagnosed food allergic patients were identified and approached, and 70 of them (85.4%) agreed to participate in our study.

### Questionnaire

A questionnaire was used to characterize the food allergies and to assess knowledge, attitudes, and practices related to food allergies and allergens among Lebanese food handlers. The questionnaire was developed based on the one used in 2 similar studies.^7^^,^^8^ It was translated to Arabic by a sworn translator and back translated to English to validate the translation. Appropriate modifications were made to some questions in terms of cultural specificity. The questionnaire was composed of 3 sections and 49 questions. It was piloted to test its clarity and the average time needed to complete it. The first section of the questionnaire was related to the demographic characteristics of the participants. The second section assessed knowledge regarding food allergies and allergens, while the third section was about food allergies and allergens attitude and practices. Approval of the Institutional Review Board at our university was obtained prior to approaching the participants.

### Statistical analysis

All data were coded and entered into SPSS V26 (IBM Corporation, Armonk, NY). Categorical data were summarized using frequency and percentage, whereas numerical data were summarized using mean and standard deviation. Scores for knowledge and best practice were calculated using summation of items then transformation into scores over 100, and were tested for normal distribution. Pearson's correlation was used to correlate knowledge score with best practice score. Differences in means were tested using the independent samples t-test and p-values were evaluated at the 0.05 significant level.

## Results

### Characteristics of the study population

A total of 70 valid questionnaires were included in the study. Females outnumbered males (81.4% vs. 19.6%). Females suffer more frequently from food-related symptoms than males due to effects of hormone, gender-specific behavior, risk perception, or medications intake.^9^ In addition, 62.9% of participants were below 45 years of age, 72.9% holders of a university degree, 30% having a health related educational background, and 58.6% living in the capital Beirut. In terms of their medical history, 70.7% reported that were diagnosed using blood test (a total serum IgE or an allergen-specific IgE). Furthermore, 54.4% had their first allergic reaction before the age of 21, 54.3% were diagnosed before the age of 21, and 21.4% have immediate family members diagnosed with food allergies.

### Characteristics of the food allergies and allergens

The top 3 were wheat, shellfish, and fish. Shrimp was the mostly reported among the shellfish, tuna among the fish, eggplant among vegetables, almonds among tree nuts, strawberry among fruits, and pepper among spices. In terms of food allergy reactions, itchy skin, and rash were mostly reported (71.4% and 68.6%, respectively). Within the past year of the survey filling date, 52 (74.3%) participants reported no food allergic reactions, and 67 (95.7%) did not miss any working days due to their food allergy, and only 1 (1.4%) visited the emergency room. On the other hand, 48 (68.6%) visited the emergency room at least once in their lifetime, and 6 (8.6%) only ever participated in a food allergy treatment.

### Knowledge and practices related to food allergies and allergens

To assess the knowledge of the diagnosed food allergic participants of our study, 17 true or false questions were asked. Average knowledge score of participants was calculated to be 84.2 ± 11.5% (Table 1). Twenty-one (30.0%) of the participants scored above 90%, 26 (37.1%) scored 80–89%, 17 (24.3%) scored 70–79%, while 6 (8.5%) scored less than 69%. To assess the practices of the participants of our study, 7 questions were asked. Average best practices score of participants was calculated to be 47.8 ± 28.3% (Table 1). Thirteen (18.6%) of the participants scored above 80%, 11 (15.7%) scored 70–79%, while 45 (65.7%) scored less than 69%.

**Table 1:** Responses and mean score of food allergies and allergens knowledge and practices questions

KNOWLEDGE Question	Correct Answer (*T: true/F; false*)	Correct	
		N	%
Individuals with food allergies can safely consume a small amount of that food	F	69	98.60%
Cooking stops food from causing allergies	F	66	94.30%
If a food allergic person is having an allergic reaction, s/he should be served cold water to dilute the allergen	F	62	88.60%
Removing an allergen from a meal after cooking makes the meal safe	F	64	91.40%
A food allergy is an abnormal response of the immune system to an ordinarily harmless food or ingredient in a food	T	67	95.70%
A food allergy is not common but can be fatal	T	61	87.10%
Children may outgrow some food allergies	T	45	64.30%
Adults can outgrow their food allergies, especially if they stop consuming that food for a period of time	F	55	78.60%
People with allergies come mostly from families in which allergies are common	F	40	57.10%
Food allergens are usually proteins	T	49	70.00%
Lactose intolerance and milk allergy are the same condition	F	66	94.30%
Individuals who have peanut or tree nut allergy are sometimes advised to avoid both peanuts and tree nuts from their diets	T	46	65.70%
Hidden food allergens are one of the most common causes of food allergy occurrences	T	68	97.10%
Persons of a known food allergy who begin experiencing allergic symptoms while or after eating a food should initiate treatment immediately, and go to a nearby emergency room if symptoms progress	T	70	100.00%
Taking an anti-allergy medication at home or receiving a steroid injection at the pharmacy or doctor's office will stop the food allergic reaction	T	65	92.90%
Epinephrine injection in the muscle should only be given by a professional personnel, i.e. pharmacist, nurse or doctor	F	52	74.30%
Adults almost rarely outgrow their food allergy	T	57	81.40%
**Mean Knowledge score over 100 (Mean ± SD)**	**84.2 ± 11.5**		
**PRACTICE Question**	**Reported best practice**		**%**
	**N**		
Check both ingredients and precautionary information	63		90.00%
All the time check the ingredients list for any food allergens upon purchasing or before consuming any product	24		34.30%
All the time read the nutritional claims for any food allergens upon purchasing or before consuming any product	26		37.10%
All the time talk to the waiter in person to address food allergy needs and to suggest non-risky choices	38		54.30%
All the time double check with the waiter to make sure no allergens are present in your food order	32		45.70%
Carry medications to treat food allergy with you (epinephrine is the only lifesaving therapy for an anaphylactic reaction)	27		38.60%
Check if a food product bears ingredient and suitable language	24		34.30%
**Mean best practice score over 100 (Mean ± SD)**	**47.8 ± 28.3**		

## Discussion

Previous studies in the literature reported that shellfish was the most commonly reported food allergen in Mauritius^7^ and Asia.^10^ Fish is an important component of the Mediterranean diet, including the Lebanese one, due to its geographical location. Due to the availability and popularity of seafood, this may have resulted in a higher prevalence of seafood allergy, as apparent in regions such as Japan, Spain, and Philippines.^11^

Itchy skin and rash were mostly reported as food allergy reactions. The same was found in Mauritius.^7^ On the other hand, having a health related educational background resulted in a significantly (p < 0.05) higher best practices score (61%) compared to other backgrounds (42%) (Table 2). This can be explained similarly as in the knowledge score. In addition, consulting an allergist resulted in a significantly (p < 0.05) higher score (89%) (Table 2). This can be because seeing an allergist will answer all the inquiries that the patient has and increases his/her knowledge in the topic. Furthermore, participants who claimed they are knowledgeable in the topic scored significantly (p < 0.05) higher (62%) than those who did not (41%).

**Table 2:** Relations between characteristics of participants and food allergies/allergens knowledge and practice scores.

Independent variable	Knowledge score (%) (Mean ± SD)	p-value	Best practice score (%) (Mean ± SD)	p-value
**Gender**				
Male	84.62 ± 9.47	0.887	47.25 ± 28.20	0.944
Female	84.11 ± 12.02		47.87 ± 28.51	
**Age groups**				
18–34 years	84.87 ± 9.43	0.766	53.06 ± 30.40	0.945
≥35 years	83.75 ± 11.60		53.74 ± 26.69	
**Education**				
Health/Sciences	89.64 ± 11.15	**0.009**	61.22 ± 26.4	**0.008**
Others	81.87 ± 10.99		41.98 ± 27.26	
**Knowledge Perception**				
Expert/Very Knowledgeable	87.7 ± 9.42	0.086	62.34 ± 25.15	**0.003**
Somewhat/Not knowledgeable	82.6 ± 12.13		41.07 ± 27.27	
**Consult with Allergist**				
Yes	88.89 ± 10.66	**0.045**	57.14 ± 22.98	0.07
No	82.58 ± 11.47		44.51 ± 29.35	
**Affect the things you do with others**				
Great/good Deal	87.82 ± 10.94	0.192	74.49 ± 12.75	**<.001**
Moderately/little	83.3 ± 11.59		41.07 ± 27.11	
**Stress/Anxiety cause you/family**				
Great/good Deal	87.5 ± 10.60	0.084	61.31 ± 29.33	**0.003**
Moderately/little	82.48 ± 11.73		40.68 ± 25.19	

Bold p-values are significant since they are less than 0.05

When asked about whom participants consult for additional information regarding food allergies and allergens, 47 (67.1%) answered general physician. This is a higher than the one (40%) reported by Soogali and Soon (2018).^7^ On the other hand, 28 (40.0%) answered pharmacist, 18 (25.7%) answered allergist, 10 (14.3%) answered nutritionist, while only 1 (1.4%) does not consult anybody. On the other hand, when asked about which medications participants carry to treat their food allergy, 36 (51.4%) indicated that they do not, while 34 (48.6%) indicated they do. This rate is higher than the one (16%) reported by Soogali and Soon (2018).^7^ When asked about the importance of improvements that should be made, 65 (92.9%) of participants agreed that the writings on ingredients label should be in bold and have a bigger font. Mfueni et al (2018)^11^ reported that bold font was the most frequently used font when highlighting allergens in an ingredient list. Other emphasis includes color contrast, italics, or enlarged font. In Mauritius, more than 80% answered that allergens in the ingredient list should be emphasized using bold or capitalized font or highlighted with appropriate background color.^7^

In addition, 51 (73.9%) agreed that a simple language must be used. This goes in line with the results of Joshi et al (2012),^12^ who reported that complex ingredient terminology may negatively affect consumers' safety especially if they do not understand the terminologies. Misunderstanding of label terms or use of generic terms (eg, flavor or spice) were reported as the main reason for allergic reactions.

Sixty-eight (97.1%) agreed that a striking symbol to indicate the presence of allergens for illiterate people must be added vs 87.6% in Mauritius as reported by Soogali and Soon (2018).^7^ Furthermore, 63 (90.0%) agreed that allergy warnings must be placed next to ingredients list (vs. 73.5% in Mauritius as reported by Soogali and Soon (2018).^7^ Symbols such as (∗) can be used to indicate presence of allergens in food labels. Otherwise, internationally recognized symbol such as the Grossed Grain Symbol is used in Europe to imply that gluten-free products conform to safe manufacturing standards.^13^ On the other hand, 41 (58.6%) agreed that manufacturer's contact detail must be more visible (vs. more than 60% in Mauritius as reported by Soogali and Soon (2018).^7^

## Conclusion

Our study is of value to the policy makers, food industry, and healthcare practitioners as the findings represent a snapshot of the status of diagnosed food allergy status in Lebanon. Characterizing the food allergies and allergens in Lebanon will put pressure on policy makers to making allergen warning label obligatory in the country. Our results highlight the importance of ongoing educational initiatives to improve the knowledge and practices of those suffering from food allergies in the country. This conclusion goes along the findings of Hassan et al^14^^,^^15^ who assessed food safety knowledge and practices among university students and food handlers in households. Further studies looking into the knowledge and practices of food handlers in food service establishments, in addition to caregivers of allergic individuals, must also be carried out.

## Funding

No funding was provided to this study.

## Authors contribution

Dr. Hani Dimassi carried out the statistical analysis and co-wrote the manuscript. Dr. Suzan Haidar co-wrote the manuscript. Ms. Sarah Issah recruited and interviewed the participants. Dr. Hassan conceptualized the study, managed the data collection, prepared the questionnaire and co-wrote the manuscript.

## Ethics approval

Approval of the study was granted by the Institutional Review Board at the 10.13039/100010340Lebanese American University.

## Availability of data and materials

Not applicable.

## Consent for publication

All authors approved to the publication of this work.

## Declaration of competing interest

All of authors report no competing interests or financial disclosure.
